# Editorial: Molecular insights into the tumor progression of endocrine and hormone-sensitive cancers

**DOI:** 10.3389/fendo.2026.1980821

**Published:** 2026-09-08

**Authors:** Vijaya Kumar Pidugu, Hsiang-Chi Huang, Wan-Ning Li

**Affiliations:** 1 Laboratory of Cancer Biology and Genetics, Center for Cancer Research, National Cancer Institute, Bethesda, MD, United States; 2 Institute of Molecular Medicine, College of Medicine, National Cheng Kung University, Tainan, Taiwan

**Keywords:** endocrine cancer, hormone-sensitive cancer, molecular mechanisms, precision oncology, targeted therapy, tumor progression

Endocrine and hormone-sensitive cancers represent a biologically diverse group of malignancies whose development and progression are shaped by the interplay of inherited and acquired genomic alterations, dysregulated signaling, hormonal dependencies, metabolic adaptation, tumor–microenvironment interactions, and therapeutic selection pressure. Although substantial progress has been made in defining molecular drivers and developing targeted therapies, translating molecular knowledge into durable clinical benefit remains challenging. The articles assembled in this Research Topic illustrate this complexity while highlighting an important evolution in cancer research from identifying individual molecular abnormalities toward integrating genomic, metabolic, microenvironmental, and functional information to understand tumor behavior and guide precision oncology. An equally important challenge is understanding how these determinants evolve over time and contribute to changes in tumor behavior and therapeutic response.

A particularly instructive example comes from Qi et al., who investigated rare multiple endocrine neoplasia type 2A (MEN2A) families carrying the germline *RET* p.C634Y variant. Their analysis included a homozygous patient and heterozygous monozygotic twins and was complemented by a systematic review of reported homozygous *RET* mutations. Homozygous carriers showed substantially greater penetrance of medullary thyroid carcinoma (MTC) and a higher frequency of node-positive disease than heterozygous carriers. Importantly, however, genetically identical twins displayed discordant clinical and biochemical phenotypes. These observations emphasize that even a powerful oncogenic driver does not completely determine tumor behavior. Environmental exposures, developmental variation, epigenetic regulation, and other modifiers may contribute to disease expression, supporting individualized surveillance rather than assumptions based solely on genotype. Further investigation of these factors may help clarify phenotypic variability among individuals carrying the same pathogenic variant. This principle of genotype-informed but individually adapted treatment is further developed by Yang et al. in their review of molecular alterations and targeted therapy in thyroid cancer. The expanding spectrum of actionable alterations, including *BRAF* V600E, *RET* mutations and fusions, and *NTRK* fusions, has transformed the management of advanced thyroid malignancies. BRAF/MEK inhibition, selective RET and TRK inhibition, VEGFR-directed multikinase inhibitors, and emerging redifferentiation strategies demonstrate how mechanistic understanding can be translated into therapeutic selection. At the same time, co-mutations, pathway crosstalk, and acquired resistance argue against static molecular classification. Comprehensive molecular profiling, including fusion detection and reassessment as disease evolves, increasingly provides a framework for treatment sequencing and rational combination strategies. In this setting, therapeutic vulnerabilities may not remain fixed as tumors acquire new dependencies during treatment. The Research Topic also expands the concept of molecular signaling beyond classical oncogenes. Yuan et al. describe ammonia not simply as a metabolic waste product but as a biologically active component of the tumor microenvironment. Their review integrates ammonia metabolism with mTORC1 signaling, epigenetic remodeling, epithelial–mesenchymal transition, metastasis, and immune suppression, thereby illustrating how metabolic products can become signaling mediators of malignant progression. This framework is particularly relevant to treatment resistance because metabolic plasticity can allow tumors to bypass inhibition of individual pathways. Therapeutic strategies targeting ammonia production, transport, or clearance may therefore benefit from biomarker-guided patient selection could ultimately be most effective in combination with immunotherapy, targeted therapy, or other metabolic interventions. It remains unclear whether ammonia-related metabolic dependencies are stable features of tumors or emerge in response to specific microenvironmental conditions or treatment. Inherited susceptibility represents another dimension of tumor progression. Kitsera et al. characterize Ukrainian women carrying the pathogenic founder *BRCA1* c.181T>G (p.Cys61Gly) variant. Their case series demonstrates frequent early-onset breast cancer, multiple primary malignancies, marked familial clustering, and heterogeneous tumor phenotypes, including luminal-like, HER2-positive, and triple-negative disease. Thus, even within a shared hereditary predisposition, tumor biology remains heterogeneous. These findings reinforce the value of population-aware genetic testing, detailed pedigree assessment, lifelong surveillance, and risk-reduction strategies tailored to regional founder-variant landscapes.

A recurring challenge across precision oncology is that genomic information alone cannot always predict therapeutic response. Caruso et al. address this gap through patient-derived organoids (PDOs), which retain important genomic, transcriptomic, histopathological, and functional characteristics of parental tumors and enable direct pharmacological interrogation. Their review highlights applications in drug screening, radiotherapy, immuno-oncology, functional genomics, and particularly hormone-sensitive breast and prostate cancers. PDOs therefore provide a conceptual bridge between molecular precision oncology, which asks, what alteration does this tumor contain? and functional precision oncology, which asks, which treatment is this patient’s tumor sensitive to? Nevertheless, sampling bias, clonal selection, incomplete representation of stromal and immune compartments, assay variability, turnaround time, and lack of standardization remain important barriers. PDO results should consequently complement, rather than replace, genomic, pathological, and clinical information. The importance of selecting biologically faithful experimental systems is further illustrated by Padmanabhan et al. in their comprehensive review of models of high-grade serous carcinoma (HGSC) of tubo-ovarian origin. Contemporary evidence supports the distal fallopian tube epithelium as the predominant precursor site for many HGSCs, while experimental models indicate that alternative cellular origins may be possible under specific genetic contexts. The authors emphasize that no single experimental platform captures the full biology of HGSC. Cell lines, organoids, tumor slices, organ-on-chip systems, patient-derived xenografts, syngeneic models, and genetically engineered models provide complementary levels of molecular, cellular, microenvironmental, and organismal complexity. Model selection should therefore be guided by the biological question and the model’s molecular and functional fidelity to the disease being studied.

## Conclusion

Collectively, these six contributions reveal several convergent principles. Tumor progression cannot be understood through single pathways in isolation; genotype does not invariably predict phenotype; metabolism and the microenvironment actively shape malignant behavior; inherited variants require population- and patient-specific interpretation; therapeutic resistance reflects dynamic tumor evolution; and increasingly sophisticated patient-derived models are needed to translate molecular discoveries into clinically meaningful interventions.

## Future perspectives

The next phase of research should therefore integrate longitudinal genomic profiling with epigenomics, metabolomics, spatial and single-cell technologies, functional drug testing, and clinically representative models. Such multidisciplinary approaches will be essential for identifying not only molecular drivers but also the adaptive states that permit tumors to progress and escape therapy. Indeed, the ammonia-signaling review specifically points toward single-cell multi-omics, spatial transcriptomics, PDOs, and systems-level metabolic modeling as approaches capable of resolving tumor heterogeneity and improving therapeutic prediction. Similarly, PDO platforms are moving toward integration with artificial intelligence and microphysiological systems, although standardization, scalability, and prospective validation remain prerequisites for routine clinical implementation. The value of these increasingly sophisticated approaches will ultimately depend on whether they improve risk assessment, treatment selection, or the early detection of therapeutic resistance. Ultimately, the studies in this Research Topic demonstrate that precision oncology is evolving from the identification of isolated molecular targets toward a multidimensional understanding of each tumor as an evolving biological system. Connecting inherited susceptibility, oncogenic signaling, metabolism, tumor–microenvironment interactions, functional modeling, and therapeutic response will be crucial for transforming molecular insights into more precise prevention, surveillance, and treatment strategies for patients with endocrine and hormone-sensitive cancers.

